# Recent developments of phosphodiesterase inhibitors: Clinical trials, emerging indications and novel molecules

**DOI:** 10.3389/fphar.2022.1057083

**Published:** 2022-11-24

**Authors:** Andrey D. Bondarev, Misty M. Attwood, Jörgen Jonsson, Vladimir N. Chubarev, Vadim V. Tarasov, Wen Liu, Helgi B. Schiöth

**Affiliations:** ^1^ Department of Surgical Sciences, Functional Pharmacology and Neuroscience, Uppsala University, Uppsala, Sweden; ^2^ Advanced Molecular Technologies LLC., Moscow, Russia

**Keywords:** PDE inhibition, cyclic nucleotides, second messengers, sildenafil, roflumilast, apremilast, ibudilast

## Abstract

The phosphodiesterase (PDE) enzymes, key regulator of the cyclic nucleotide signal transduction system, are long-established as attractive therapeutic targets. During investigation of trends within clinical trials, we have identified a particularly high number of clinical trials involving PDE inhibitors, prompting us to further evaluate the current status of this class of therapeutic agents. In total, we have identified 87 agents with PDE-inhibiting capacity, of which 85 interact with PDE enzymes as primary target. We provide an overview of the clinical drug development with focus on the current clinical uses, novel molecules and indications, highlighting relevant clinical studies. We found that the bulk of current clinical uses for this class of therapeutic agents are chronic obstructive pulmonary disease (COPD), vascular and cardiovascular disorders and inflammatory skin conditions. In COPD, particularly, PDE inhibitors are characterised by the compliance-limiting adverse reactions. We discuss efforts directed to appropriately adjusting the dose regimens and conducting structure-activity relationship studies to determine the effect of structural features on safety profile. The ongoing development predominantly concentrates on central nervous system diseases, such as schizophrenia, Alzheimer’s disease, Parkinson’s disease and fragile X syndrome; notable advancements are being also made in mycobacterial infections, HIV and Duchenne muscular dystrophy. Our analysis predicts the diversification of PDE inhibitors’ will continue to grow thanks to the molecules in preclinical development and the ongoing research involving drugs in clinical development.

## Introduction

The cyclic nucleotide signal transduction system, collectively encompassing the 3′-5′cyclic adenosine and guanosine monophosphate (cAMP and cGMP, respectively) signaling systems, have long been a subject to intensive research for its important biological implications and associated therapeutic potential. A part of a diverse group of non-protein signaling compounds called second messengers, the cAMP and cGMP signaling systems are ultimately involved in relaying the signal produced *via* receptor-ligand interactions at the cell surface to effector proteins (Newton et al., 2016). These effector proteins, such as the cAMP and cGMP-activated protein kinases, subsequently elicit a wide variety of biological responses.

The second messengers’ intracellular concentration is maintained by complex homeostatic mechanisms. Within the cyclic nucleotide system, these include the adenylyl/guanylyl cyclase enzymes and the cyclic nucleotide Phosphodiesterase (PDE); the former is activated in response to the receptor-ligand interaction, catalyzing the conversion of AMP/GMP into 3′-5′- cAMP/cGMP, while the latter catalyzes the cyclic nucleotides’ deactivation (Newton et al., 2016). The potential of this system, especially the PDE enzymes, from the drug development point of view had been recognized in as early as of late 20th century (Amer, 1977; Pang, 1988) and there has been a continuously growing interest towards further advancement in a variety of clinical indications; according to the ClinicalTrials.gov database, there are at least 1825 recorded clinical studies involving PDE inhibitors as of 2022. However, only a relatively small number of PDE inhibitors have entered market, while several initially promising therapeutic agents proved unsuccessful (Baillie et al., 2019).

During our investigation of trends within clinical trials (Rask-Andersen et al., 2011; Rask-Andersen et al., 2014; Attwood et al., 2018), we have noticed an especially large number of studies involving both the investigational and approved agents of the PDE inhibitors class, prompting us to conduct our own detailed investigation. The aim of present analysis is to provide a comprehensive overview of the most recent trends in clinical research regarding PDE inhibitors and to speculate regarding the future of this therapeutically important enzyme family.

## The cyclic nucleotide phosphodiesterase superfamily—Overview

**FIGURE 1:**
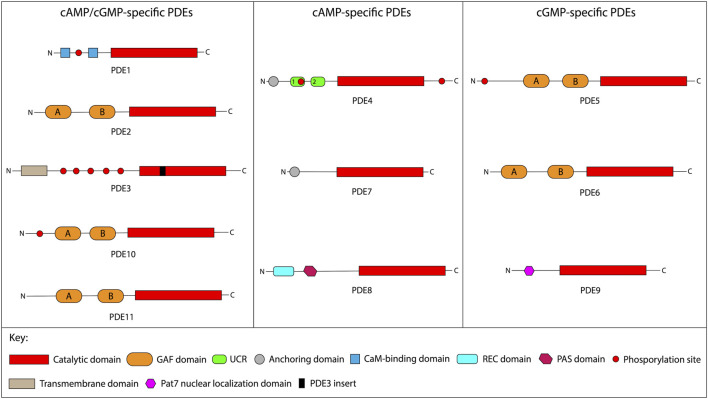
Schematic structure of PDE enzymes. This figure schematically presents principle structural features of the individual PDE isoforms. The PDE families are grouped according to the cyclic nucleotide affinities. The structural features and approximate isoform sizes of each PDE family are summarized as per (Baillie et al., 2019; Conti and Beavo, 2007; Francis et al., 2011; Maurice et al., 2014). Abbreviations: cAMP, cyclic adenosine monophosphate; cGMP, cyclic guanosine monophosphate; PDE, phosphodiesterase; GAF, cGMP-binding PDEs, Anabaena adenylyl cyclase, and Escherichia coli FhlA; UCR, upstream conserved region; CaM; calmodulin; REC, signal regulatory domain; PAS, Per-ARNT-Sim; Pat7, 7-residue nuclear localization signal.

The cyclic nucleotide PDEs is a diverse superfamily of 11 families, encoded by 21 genes and responsible for catalyzing the 3′-cyclic phosphate bond hydrolysis in the cyclic nucleotide molecules, resulting in inactive compounds (Francis et al., 2011a). Structurally, the PDE enzymes are characterized by the conserved C-terminal catalytic domain and diverse N-terminal regions, comprised of several subdomains; the family designation is based on the C-terminal catalytic domain homology (Francis et al., 2011a). Each of the individual PDE families is characterized by varying specificities towards the cyclic nucleotide substrate: the families 1, 2, 3, 10, and 11 hydrolyze both cAMP and cGMP with comparable affinity; the families 4, 7, and 8 exhibit higher affinities towards cAMP, while the families 5, 6, and 9 are more specific towards cGMP (Francis et al., 2011a). The expression pattern among different PDE families is typically broad and characterized by predominantly cytosolic intracellular localization (Bender and Beavo, 2006; Conti and Beavo, 2007). The Figure 1 summarizes the known evidence regarding the structural and functional features of each family, while Table 1 illustrates the isoforms and expression patterns.

**TABLE 1:** PDE isoforms and expression pattern. This table provides a summary on the PDE enzymes’ isoforms and their expression pattern.

PDE family	PDE isoforms	Expression pattern
PDE 1	PDE 1A, 1B, 1C	Nervous system (Essayan, 2001)), cardiovascular system, lungs, immune system (Yan et al., 2001), male reproductive system (Shakur et al., 2001; Vasta et al., 2005)
PDE 2	PDE 2A	Adrenal medulla, brain, heart, lung, liver, platelets, macrophages (Yan et al., 2001; Bender and Beavo, 2006)
PDE 3	PDE 3A, 3B	Platelets, lung, liver, germinal neuroepithelium, mature neurons, adipose tissue, β-cells of the pancreas, renal collecting duct epithelium, T-lymphocytes, macrophages, oocytes and developing spermatocytes (Davis et al., 1989; Yan et al., 2001)
PDE 4	PDE 4A, 4B, 4C, 4D	Nervous system (Salanova et al., 1999), male reproductive system (Richter et al., 2011), inflammatory (Yan et al., 2001) and cardiovascular (Richter et al., 2005; Bender and Beavo, 2006) systems, kidney and liver (Coquil et al., 1980; Yan et al., 2001)
PDE 5	PDE 5A	Platelets, lungs (Francis et al., 1980; Kotera et al., 2000), brain, kidney, pancreas (Smith et al., 2003), the vascular smooth muscle, heart, placenta, skeletal muscle, liver, gastrointestinal tissues and immune system (Yan et al., 2001; Bender and Beavo, 2006)
PDE 6	PDE 6A, 6B, 6C, 6D, 6G, 6H	Retinal photoreceptors (Bender and Beavo, 2006)
PDE 7	PDE 7A, 7B	Immune system (Smith et al., 2003), skeletal muscle, heart (Han et al., 1997), brain, liver, kidney and pancreas (Gardner et al., 2000; Sasaki et al., 2000)
PDE 8	PDE 8A, 8B	Testis, ovaries, spleen, small intestine, colon, kidney, immune system, brain and thyroid gland (Yan et al., 2001; Bender and Beavo, 2006)
PDE 9	PDE 9A	Kidney, brain, spleen, prostate, the intestines, lung and liver (Bender and Beavo, 2006)
PDE 10	PDE 10A	Nervous system, testis, thyroid and pituitary glands, and muscle tissues (Bender and Beavo, 2006)
PDE 11	PDE 11A	Skeletal muscle, male reproductive system, pituitary and salivary glands, liver, heart and kidney (Bender and Beavo, 2006)

Biological functions of the individual families are diverse, including involvement in such processes, as myocytes contractility, male and female sex cells development and functioning, inflammatory cells activation, steroidogenesis or neuronal signaling (Bender and Beavo, 2006; Tsai and Beavo, 2011; Maurice et al., 2014). Such a variety of physiological involvement inevitably suggests involvement in human pathology as well. Indeed, numerous reviews have showed evidence on PDE-mediated implications in such diseases, as cancer (Peng et al., 2018), neurologic (Halene and Siegel, 2007; Nthenge-Ngumbau and Mohanakumar, 2018; Gurney, 2019), inflammatory (Page and Spina, 2011), pulmonary (Fan Chung, 2006; Mokry et al., 2018; Joskova et al., 2020; Kawamatawong, 2021; Mokra and Mokry, 2021), pediatric (Mokra et al., 2018), and cardiovascular disorders (Ravipati et al., 2007), among others. Additionally, the PDE enzymes are characterized by several factors, including a high degree of isoform variation, a high degree of isoform specificity towards substrates, distinct tissue expression and subcellular localization patterns among different isoforms, and the generally favorable intracellular cyclic nucleotides’ concentration, that further highlight their potential as drug targets (Bender and Beavo, 2006).

The global efforts to exploit the PDE enzymes therapeutically have so far produced a large number of molecules with varying selectivity and therapeutic applications, as well as a growing number of agents under development (Baillie et al., 2019). In the next section, we will provide an introduction to the PDE inhibitors as a class of therapeutic agents, as well as discuss a brief methodology of the dataset, used in our study.

## Phosphodiesterase inhibitors overview and dataset

Chemically, the PDE inhibitors predominantly belong to a broad range of nitrogen-containing classes (Weinryb et al., 1972; Weishaar et al., 1985; Dyke and Montana, 2002; Andersson, 2018; Zagorska, 2020). At least one non-nitrogenous compound, an isoflavone derivative genistein, has also been identified to affect the PDE enzymes (Nichols and Morimoto, 2000). The majority of marketed PDE inhibitors are characterized by non-selective action; however, the PDE 3 and 5-selective inhibitors represent a notable portion of the PDE inhibitors market (Baillie et al., 2019).

The earliest identified class of PDE inhibitors is the class of xanthine derivatives, which provided important tools for the further development of this therapeutic class; acting as competitive inhibitors, these compounds are characterized by structural features that resemble the purine moiety of cyclic nucleotides: the heterocyclic ring which is comprised of a six-membered pyrimidine ring, conjoined with a five-membered ring containing nitrogen atoms (Francis et al., 2011b). The earliest such compounds, caffeine and theophylline, were both characterized by non-selective and relatively weak inhibitory activity (Choi et al., 1988); however, alkylxanthine compounds, such as 1-methyl-3-isobutylxanthine, were shown to be up to 15-times more potent as compared to theophylline (Beavo et al., 1970). These discoveries, subsequently, have resulted in the emergence of an ever-growing range of improved molecules, including the PDE 5-selective sildenafil and related compounds.

**FIGURE 2:**
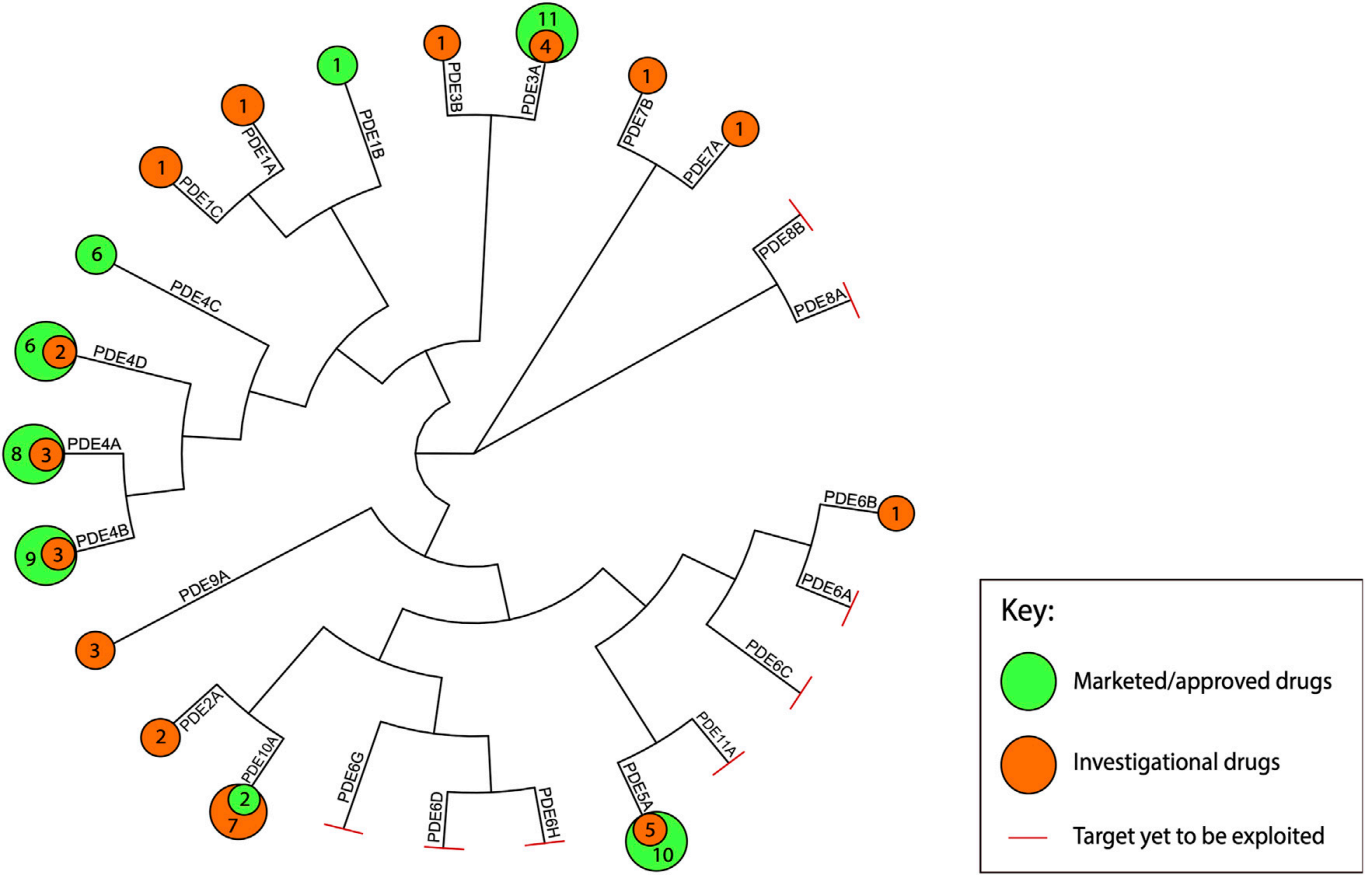
Marketed and investigational PDE inhibitors mapped to the human PDE proteins. This figure illustrates the phylogenetic relationship between the human PDE isoforms and maps the identified marketed and investigational agents to their targets. The amino acid sequences of each for each of the human PDE proteins were obtained from UniProt (UniProtKB database) (UniProt Consortium, 2021). Multiple sequence alignment was performed using Clustal Omega (RRID:SCR_001591). The phylogenetic tree was visualized in R Studio (Paradis and Schliep, 2019; Yu, 2020) and annotated in Adobe Illustrator CC 22.1.

In our dataset, we have included 87 unique PDE inhibitors. The Figure 2 maps the approved and investigational inhibitors to individual PDE isoforms, as well as provides the phylogenetic relationship between PDE proteins. The data was extracted from updated versions of our previously published analyses on drug-target interactions of both (The US Food and Drug Administration TUS, 2018) approved agents as well as agents in clinical development (Rask-Andersen et al., 2011; Rask-Andersen et al., 2014; Attwood et al., 2018). These studies were originally based on information from the Drugs in Clinical Trials Database (discontinued) from CenterWatch^1^ and the DrugBank database (Wishart et al., 2018) and spans from 1983 to 2019. The targets, mechanisms of action, and indications were manually assessed using published studies and public databases including Drugs@FDA^2^, EMA (European Medicines Agency. 2019a) Medicines^3^ and NICE (The National Institute of Health. 2022) Technology appraisal guidance^4^ databases. US clinical trial information was obtained from the National Institute of Health Clinical Trials resource^5^. Where applicable, EU clinical trial information, identified through the EMA EU Clinical Trials Register (European Medicines Agency, 2019b)^6^, was additionally included. To ensure our review is as comprehensive as possible in regards of the recent advances, we additionally reference reviews on PDE inhibitors in clinical trials and/or selected diseases published throughout the past 10 years (i.e., 2010–2020) (Page and Spina, 2011; Matera et al., 2014; Tobin, 2015; Drobnis and Nangia, 2017; Geerts et al., 2017; Knott et al., 2017; Peng et al., 2018; Giorgi et al., 2020; Tzoumas et al., 2020).

In sections below, we will discuss various aspects of the PDE inhibitors’ clinical uses and research. For the approved agents, we will briefly discuss the rationale behind employing PDE inhibitors in the relevant disorders and discuss the efficacy and safety profiles of the included drugs. For the investigational agents, we will provide a summary on some of the currently available clinical and/or pre-clinical data, and will comment on the future of this important class of therapeutic agents, highlighting its currently evident strengths and weaknesses.

## Phosphodiesterase inhibitors in clinics—Current status

As of 2022, we identified 35 agents that have been approved and authorized for marketing by either FDA or any other drug regulatory authority. The approval statuses and relevant trade names were determined through the DrugBank, Drugs@FDA and EMA Medicines databases records, Google search engine, or cited as per (Baillie et al., 2019). The majority of marketed indications include respiratory and cardiovascular diseases, as well as several inflammation-mediated pathologies of skin and joints. Central and peripheral nervous system disorders are also represented. Tables 2–Tables 4 summarize the drugs, their chemical structures, PDE isoform selectivity and indications.

**TABLE 2:** Marketed PDE inhibitors in pulmonary diseases. This table provides a summary on the available clinical evidence for marketed PDE inhibitors in lung diseases. Trade names, developer name and the targeted PDE isozymes are listed according to the PubChem, DrugBank and Drugs@FDA database records. Chemical structures are derived from the PubChem database (Kim et al., 2021).

Drug name	Chemical structure	Trade name(s)	Developer	PDE selectivity	Indication
Theophylline	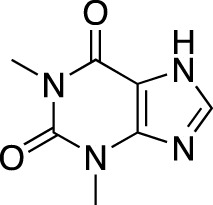	Theolair^®^, Slo-bid^®^, Theo-dur^®^	Multiple	Non-selective	Asthma, chronic obstructive pulmonary disease
Aminophylline	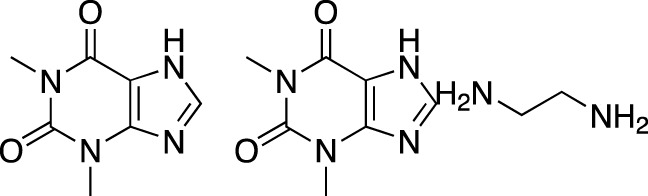	Phyllocontin^®^	Multiple	Non-selective	Asthma, chronic obstructive pulmonary disease
Dyphylline	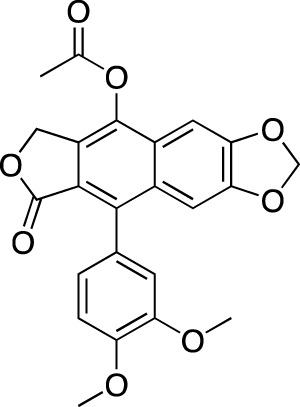	Dilor^®^, Lufyllin^®^, Protophylline^®^	Multiple	Non-selective	Asthma, chronic obstructive pulmonary disease
Oxtriphylline	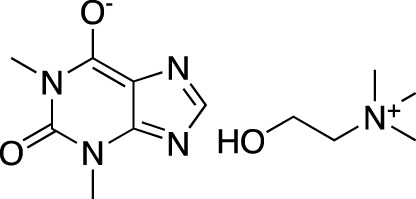	Choledyl^®^	Multiple	Non-selective	Asthma, chronic obstructive pulmonary disease
Roflumilast	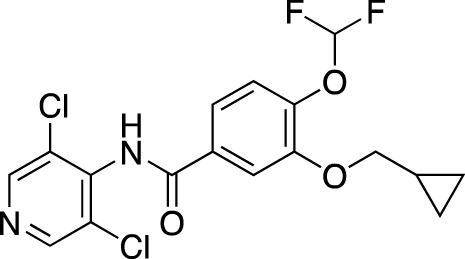	Daliresp^®^, Daxas^®^	ALTANA Pharma	PDE 4	Chronic obstructive pulmonary disease
Ibudilast	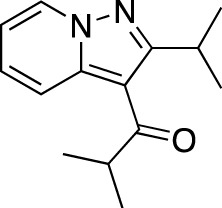	Ketas^®^, Pinatos^®^	Kyorin Pharmaceutical	PDE 3A; PDE 4	Asthma
Enprofylline	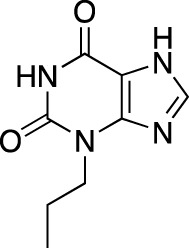	Nilyph^®^	Draco Lakemedel/Merck and Co.	Non-selective	Asthma

### Phosphodiesterase inhibitors in respiratory diseases

Obstructive respiratory diseases (OPD), such as asthma or the chronic obstructive pulmonary disease (COPD), have been one of the earliest marketed indications, associated with the PDE inhibitors. Asthma and COPD are common heterogeneous, typically inflammation-mediated diseases of the airways, characterized by the airflow limitation (Rabe and Watz, 2017; Papi et al., 2018). Non-selective theophylline and its derivatives aminophylline, dyphylline and oxtriphylline had been widely used in managing bronchospasm in asthma and COPD under multiple trade names (Table 2); xanthines are characterized by multiple mechanisms of action, including adenosine receptor antagonism, phosphoinositide-3-kinase inhibition and histone deacetylase activation (Spina and Page, 2017). Other marketed PDE inhibitors in respiratory disorders include PDE 4-selective roflumilast, approved by the FDA (Daliresp^®^, 2011) and EMA (Daxas^®^, 2010) in COPD; PDE 4-selective ibudilast, marketed in Japan and several other Asian markets for the use in asthma as Ketas^®^ and Pinatos^®^, and non-selective enprofylline, marketed in asthma as Nilyph^®^.

The rationale behind targeting PDEs to manage the airway diseases is based on the involvement of several families in the inflammatory and structural cells within the respiratory system. Among the individual PDE families, PDE 3, PDE 4, PDE 5, PDE 7, PDE 8, and PDE 9 have been described to hold the highest degree of relevance (Ntontsi et al., 2019). Inhibiting these enzymes result in a variety of cellular responses, including the airway smooth muscle relaxation, bronchodilation and inhibition of inflammatory pathways. Particularly, PDEs 4 and 5 inhibition has also been described to affect the process of airway wall remodeling (Fan Chung, 2006).

In the clinical setting, the evidence is scarce and limited to PDE 4 inhibitors in COPD, showing a benefit in improving lung function and reducing the likelihood of exacerbations, but without a significant impact on the quality-of-life, based on the results of a systematic review of clinical trials involving cilomilast, roflumilast, and tetomilast (Janjua et al., 2020). A 1-year study of roflumilast in severe COPD has specifically shown a post-bronchodilator forced expiratory flow increasing by 39 ml versus placebo without significant changes in the COPD exacerbation rate (mean values 0.86 vs. 0.92 exacerbations per patient with roflumilast vs. placebo) (Calverley et al., 2007). However, a subset of patients in stage IV disease has shown a greater decrease in the rate (1.01 vs. 1.59 exacerbations per patient). The common adverse drug reactions (ADRs) in PDE 4 inhibitors include diarrhea, nausea, vomiting, dyspepsia, and headache (Janjua et al., 2020). Roflumilast, in particular, is described to be additionally associated with insomnia, weight loss and depressed mood. In asthma, the sole published evidence on ibudilast has shown an improvement in airway hypersensitivity and reduction in asthma attack severity (Kawasaki et al., 1992).

To summarize, PDE inhibitors are currently recognized as an add-on therapy in patients with COPD showing persistent symptoms or exacerbations (Janjua et al., 2020). The major obstacles in their wider clinical use and development are safety profile negatively affecting compliance in patients with COPD and lack of clinical data in patients with severe asthma (Rabe and Watz, 2017). A currently Recruiting Phase 1 trial of roflumilast in patients with severe asthma (NCT04108377) could provide further insights on the clinical applicability of PDE 4 in asthma. Additionally, two novel PDE inhibitors, ensifentrine, and tanimilast, are currently in clinical trials for COPD and asthma; ClinicalTrials.gov records mention two Recruiting Phase 3 studies of tanimilast in COPD (NCT04636814, NCT04636801), while ensifentrine is in two Ongoing Phase 3 trials in COPD and a Phase 2 study in asthma that has shown a dose-dependent bronchodilation similar to salbutamol, but without impacting potassium levels and having a less significant impact on heart rate and pulse rate (Bjermer et al., 2019).

### Phosphodiesterase inhibitors in cardiovascular diseases

Some of the currently most widely marketed PDE inhibitors fall under the diverse category of cardiovascular drugs, primarily including cardiotonics, vasodilatory agents, as well as antiaggregants. Cardiovascular diseases (CVD) are a heterogeneous group of disorders affecting heart and blood vessels; the (World Health Organization, 2022) includes coronary heart disease, cerebrovascular disease, peripheral arterial disease, rheumatic heart disease, congenital heart diseases and venous thromboembolism into the term. The approved indications in this category include erectile dysfunction (ED), congestive heart failure (CHF), intermittent claudication, hypertension, and thrombosis-related complications (Table 3).

**TABLE 3:** Marketed PDE inhibitors in cardiovascular diseases. This table provides a summary on the available clinical evidence for marketed PDE inhibitors in cardiovascular diseases. Trade names, developer name and the targeted PDE isozymes are listed according to the PubChem and DrugBank database records. Chemical structures are derived from the PubChem database (Kim et al., 2021).

Drug name	Chemical structure	Trade name(s)	Developer	PDE selectivity	Indication
Dipyridamole	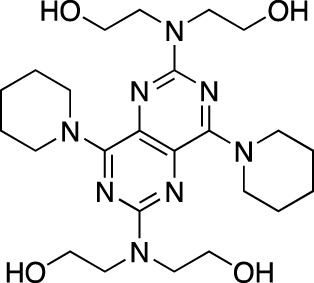	Persantine^®^	Boehringer Ingelheim	PDE 4A; PDE 5; PDE 10	Postoperative thromboembolic complications prevention
		Aggrenox^®^ (w/aspirin)			Stroke prevention
Amrinone	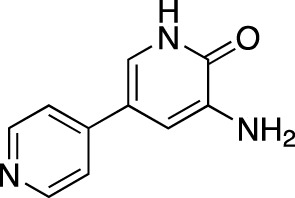	Inocor^®^	Sanofi Aventis	PDE 3A, PDE 4	Congestive heart failure
Pentoxifylline	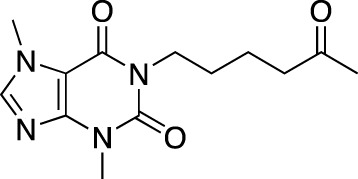	Trental^®^, Pentoxil^®^	Sanofi Aventis	Non-selective	Intermittent claudication
Cicletanine	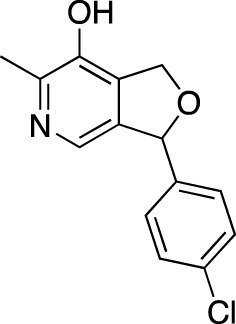	Tenstaten^®^	Ipsen	PDE 5; PDE 9	Hypertension
Olprinone	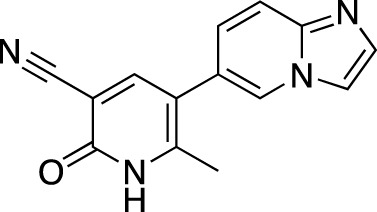	Coretec^®^	Eisai	PDE 3	Heart failure
Enoximone	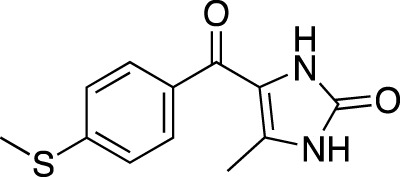	Perfan^®^	Sanofi Aventis	PDE 3A	Congestive heart failure
Milrinone	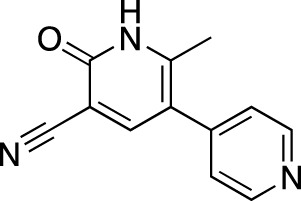	Primacor^®^, Corotrope^®^	Baker IDI/Hyloris Pharmaceuticals	PDE 3A; PDE 4A	Congestive heart failure
Anagrelide	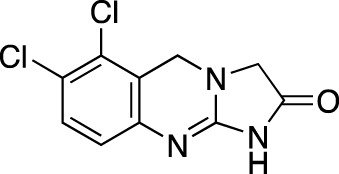	Agrilyn^®^, Xagrid^®^	Takeda	PDE 3A; PDE 4B	Secondary thrombocytopenia, thrombosis-related events
Sildenafil	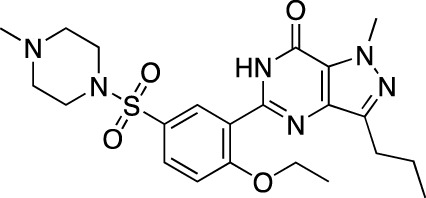	Viagra^®^	Pfizer	PDE 5	Erectile dysfunction
		Revatio^®^			Pulmonary arterial hypertension
Cilostazol	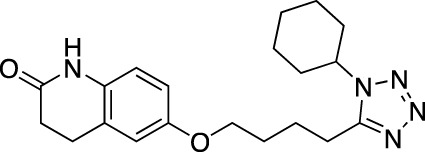	Pletal^®^	Otsuka Pharmaceutical	PDE 3A; PDE 4A	Intermittent claudication
Tadalafil	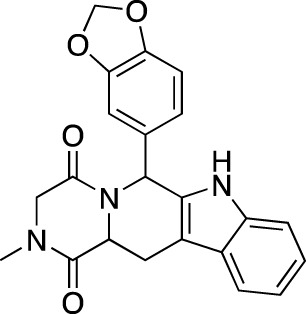	Cialis^®^	GlaxoSmithKline	PDE 5	Erectile dysfunction
		Adcirca^®^			Pulmonary arterial hypertension
Vardenafil	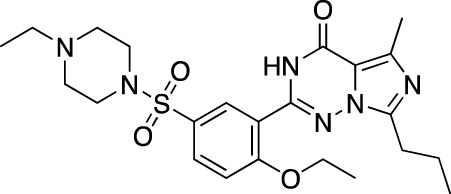	Levitra^®^, Vivanza^®^, Staxyn^®^	Bayer	PDE 5	Erectile dysfunction
Udenafil	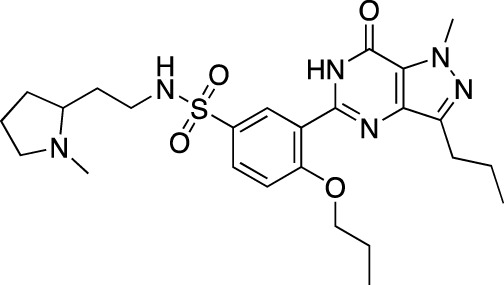	Zydena^®^	Dong-A Pharmaceutical	PDE 5	Erectile dysfunction
Mirodenafil	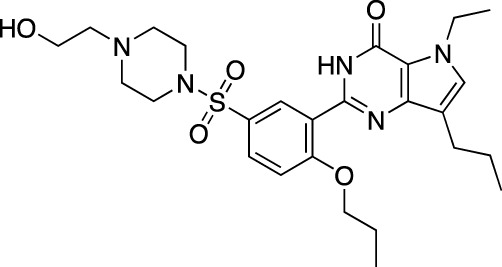	Mvix^®^	SK Chemicals	PDE 5	Erectile dysfunction
Avanafil	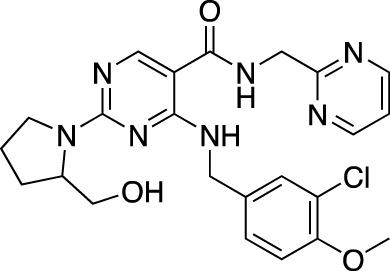	Stendra^®^, Spedra^®^	Tanabe Seiyaku	PDE 5	Erectile dysfunction
Lodenafil	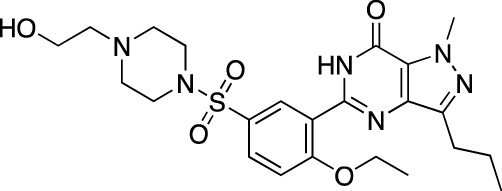	Helleva^®^	Cristalia	PDE 5	Erectile dysfunction
Papaverine	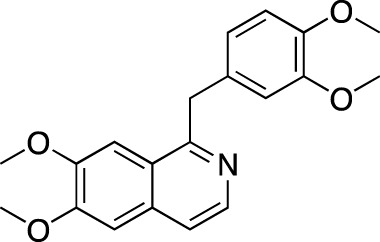	Pavabid^®^, Pavacen^®^, Pavagen^®^	Multiple	PDE 4B; PDE 10	Erectile dysfunction, spasm-induced cerebral and peripheral ischemia
Ibudilast	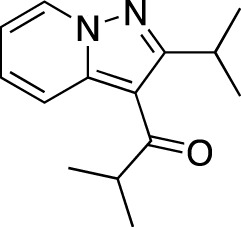	Ketas^®^, Pinatos^®^	Kyorin Pharmaceutical	PDE 3A; PDE 4	Post-stroke dizziness
Vinpocetine	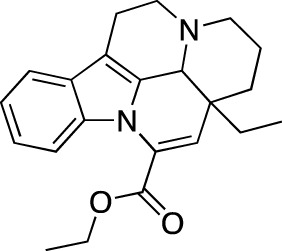	Cavinton^®^	Multiple	PDE 1	Cerebrovascular disorders; dietary supplement
Pimobendan	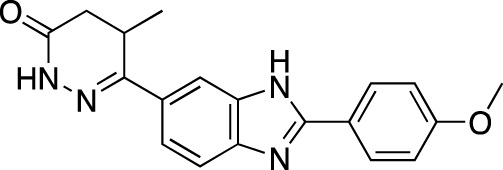	Acardi^®^, Vetmedin^®^	Boehringer Ingelheim	PDE 3	Heart failure in veterinary practice

The earliest FDA-approved^7^ cardiovascular PDE inhibitors are PDE 3-selective dipyridamole (Persantine^®^, 1961), amrinone (Inocor^®^, 1984) and milrinone (Primacor^®^, 1987), and non-selective pentoxifylline (Trental^®^, 1984). Dipyridamole is approved as an adjunct to coumarin anticoagulants in the prevention of postoperative thromboembolic complications of cardiac valve replacement; in combination with aspirin (Aggrenox^®^, 1999), it is indicated for the stroke risk reduction. Amrinone and milrinone are both marketed in CHF, while pentoxifylline is approved in intermittent claudication. Other identified FDA approvals within this category include PDE 3-selective anagrelide (Agrylin^®^, 1997; also authorized as Xagrid^®^ by the EMA in 2004), PDE 5-selective sildenafil (Viagra^®^, 1998) and its derivatives avanafil (Stendra^®^, 2012; also authorized as Spedra^®^ by the EMA in 2013), tadalafil (Cialis^®^, 2003) and vardenafil (Levitra^®^, 2003 and Staxyn^®^, 2010; also authorized as Vivanza^®^ by the EMA in 2003), and PDE 3-selective cilostazol (Pletal^®^, 1999). Anagrelide is approved for treating thrombocythemia, secondary to myeloproliferative malignancies, as well as to reduce risks of thrombosis-related events. Sildenafil, along with avanafil, tadalafil, and vardenafil, are all marketed in ED; sildenafil and tadalafil are also marketed in pulmonary arterial hypertension as Revatio^®^ (2005) and Adcirca^®^ (2009), respectively. Cilostazol is approved in intermittent claudication. Additionally, a number of PDE inhibitors, such as cGMP-PDE-specific cicletanine, PDE 3-selective enoximone and olprinone, non-selective papaverine, ibudilast, and PDE 5-specific mirodenafil, udenafil, and lodenafil, have received marketing authorization in other regions. Cicletanine is authorized in France for the treatment of hypertension (Tenstaten^®^, 1986). Enoximone is marketed across Europe for the treatment of CHF, initially authorized in France (Perfan^®^, 1987); olprinone is authorized for the same indication in Japan (Coretec^®^, 1986). Papaverine is another PDE inhibitor, used in ED, as well as vascular spasm-associated cerebral and peripheral ischemia; it is marketed worldwide under various trade names (Table 2). Ibudilast is marketed in post-stroke dizziness (Ketas^®^ and Pinatos^®^). Mirodenafil (Mvix^®^, 2007) and udenafil (Zydena^®^, 2005) are both approved in the Asian markets for the treatment of ED. Lodenafil (Helleva^®^) is marketed for ED in Brazil. PDE 1-specific vinpocetine (Cavinton^®^) is marketed as a dietary supplement in managing cerebral vascular disorders and cognitive impairment. At least one cardiovascular PDE inhibitor is also used in veterinary practice; PDE 3-specific pimobendan is marketed under various trade names, including Acardi^®^ (1997) for treatment of CHF in dogs.

The cardiac PDE families include PDEs 1, 2, 3, 4, 5, 8, 9, and 10; dysregulation in their expression patterns, activation and subcellular localization is often associated with CVDs (Chen and Yan, 2021). The bulk of currently marketed agents target PDEs 3 and 5, with PDEs 1, 4, 9, and 10 being additional targets. The PDE 3 inhibition was initially found to regulate the contractile function of the heart; global PDE 3A knockout increases cardiac contractility and relaxation through cAMP-dependent elevations of Ca^2+^ transient amplitudes and Ca^2+^ contents in the sarcoplasmic reticulum (Sun et al., 2007), which subsequently became the theoretical basis for its usage in congestive heart failure. Another important effect of PDE 3 is on the cardiomyocyte survival; chronic inhibition of its activity or reduction of PDE 3A expression induces apoptosis associated with a persistent induction of inducible cAMP early repressor (ICER), while preventing PDE 3A reduction is able to disrupt the PDE 3A-ICER feedback loop and protect cardiomyocytes from apoptosis (Ding et al., 2005). Furthermore, PDE 3 inhibition stimulates vascular relaxation, reducing peripheral and pulmonary vascular resistance and enhancing coronary blood flow (Chen and Yan, 2021). The PDE 5 inhibition has been shown to exert cardioprotective effects; a study involving sildenafil and vardenafil on a rabbit model of ischemia has shown a mitochondrial ATP-sensitive K channel opening-dependent protective effect against reperfusion injury (Ockaili et al., 2002). Additionally, studies involving sildenafil have showed a RhoA/Rho-kinase pathway inhibition-mediated attenuation of the left ventricular dysfunction (Chau et al., 2011) and the inhibition of the hypertrophy progression, fibrosis, and chamber remodeling, also improving basal and β-stimulated contractility and relaxation *via* protein kinase G activity, as well as Ca^2+^ handling (Nagyama et al., 2009).

Clinically, the bulk of published evidence is on the PDE 5 inhibitors. In ED, these agents are the first-line treatment in patients with primary disease with efficacy relating to placebo ranging from 0.47 in sildenafil to 0.26 in mirodenafil, based on the results of a trade-off network meta-analysis of 82 trials (*n* = 47,626 patients) (Chen et al., 2015). Vardenafil and lodenafil both have the relative efficacy of 0.35, udenafil and tadalafil—0.33 and avanafil—0.29. The most common ADRs, according to the analysis of 72 trials (*n* = 20,325) (Chen et al., 2015), are headache, flushing, dyspepsia, and nasal congestion; the frequency at therapeutic doses is highest in sildenafil (18.42%) and lowest in mirodenafil (10.23%). A recent meta-analysis of 44 trials (*n* = 3,853 patients) (Mykoniatis et al., 2021) has shown that combining PDE 5 inhibitors with other interventions, such as antioxidants, daily tadalafil, shockwaves or a vacuum device, improves the outcome, especially in the refractory and/or hard-to-treat disease. In pulmonary hypertension, a systematic review of 36 trials (*n* = 2,999 patients) (Barnes et al., 2019) has shown that treatment with PDE 5 inhibitors is associated with an improvement in WHO functional class and 6-min walking distance (up to 48 m), as well as reduced mortality, in patients with Group 1 pulmonary arterial hypertension.

PDE 3 inhibitors are another class of CVD-active inhibitors that has been explored clinically. In CHF, milrinone is described as an important option to treat patients with refractory disease (Chen and Yan, 2021); it functions by improving cardiac contractility and relaxation, induces vasodilation and has the overall effect of increased cardiac output, improvement of left ventricle-arterial coupling and enhanced cardiac mechanical efficiency (Ayres and Maani, 2022). ADRs associated with milrinone, such as an increased risk of arrhythmias and hypotension, are often dose-limiting, however (Chong et al., 2018). In intermittent claudication, according to the results of a systematic review of 16 trials (*n* = 3,972 patients) (Brown et al., 2021), the use of cilostazol is associated with improvements in functional status (mean initial claudication distance (ICD) of 26.49 m higher in cilostazol group vs. placebo group) and quality-of-life; comparison with pentoxifylline did not show any differences in functional status (mean ICD of 20 m higher in cilostazol group vs. pentoxifylline group). In cilostazol, the common ADRs are headache, diarrhea, dizziness, pain, and palpitations, headache being the most common (Brown et al., 2021). In thrombocytopenia, anagrelide is used in essential thrombocytopenia typically as a second-line therapy, preferentially in younger patients (Birgegård, 2016). A recently published longitudinal study (*n* = 150 patients) (Mazzucconi et al., 2020) have showed a response rate of 85.4%; ADRs included palpitations, peripheral vasodilation, anemia, diarrhea, gastric distress and thrombotic events, suggesting the importance of assessing thrombotic risk and monitoring cardiac function. The drug was also found to be characterized by nephrotoxicity, increasing the risk of renal function impairment, according to a recent retrospective study (Kwiatkowski et al., 2021).

Here, PDE inhibitors have been shown to be valuable tools to improve the functional status in patients with vascular and CVDs. It is suggested that developing isoform-specific inhibitors and/or activators could improve the clinical value of PDE modulators in CVDs, while modulating isoform-specific protein-protein interactions within specific signalosome could provide a strategy to improve the molecules’ specificity (Chen and Yan, 2021). In HF, an interesting novel approach is PDE 1 inhibition, investigated with ITI-214 (lenrispodun); it was shown to induce inodilator effects after a single oral dose, increase mean left ventricular power index and cardiac output, and reduce systemic vascular resistance (Gilotra et al., 2021).

### Phosphodiesterase inhibitors in inflammatory disorders

Several PDE inhibitors have been marketed in inflammatory disorders affecting the skin and joints (Table 4). The FDA approvals in this category include PDE 4-specific amlexanox (Aphthasol^®^, 1996), apremilast (Otezla^®^, 2014), and crisaborole (Eucrisa^®^, 2016). Amlexanox was originally approved for the treatment of recurrent aphthous ulcerations in the oral mucosa; however, current FDA records list the drug as discontinued, presumably due to termination of licensing agreement between the drug’s developer, ULURU Inc., and Discus Dental Inc., the company that held exclusive rights for sales and marketing in the United States. Apremilast is approved for the treatment of active psoriatic arthritis and moderate-to-severe plague psoriasis, and crisaborole is approved in mild-to-moderate atopic dermatitis. Ibudilast, in addition to its previously mentioned uses in asthma and post-stroke dizziness, is marketed for allergic conjunctivitis as Ketas^®^ and Eyevinal^®^. Recently, difumilast was marketed in Japan as Moizerto^®^ (2021).

**TABLE 4:** Marketed PDE inhibitors in inflammatory and nervous system disorders. This table provides a summary on the available evidence for marketed PDE inhibitors in inflammatory diseases and some neurologic pathologies. Trade names, developer and the targeted PDE isozymes are listed according to the PubChem and DrugBank database records. Chemical structures are derived from the PubChem database (Kim et al., 2021).

Drug name	Chemical structure	Trade Name(s)	Developer	PDE selectivity	Indication
Amlexanox	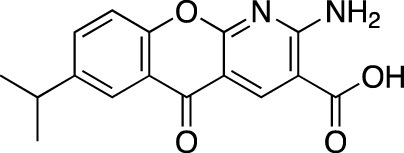	Aphthasol^®^	Takeda	PDE 4	Aphthous ulcers
Apremilast	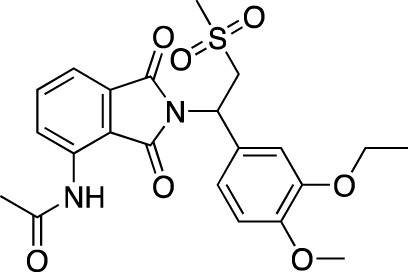	Otezla^®^	Celgene	PDE 4	Psoriatic arthritis, plague psoriasis
Caffeine	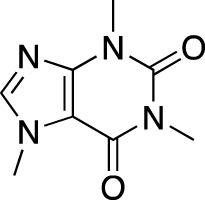	Cafcit^®^	Multiple	PDE 4B	Apnea of prematurity
Crisaborole	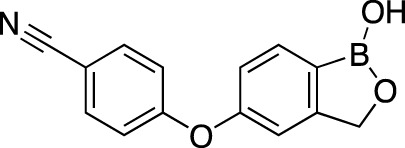	Eucrisa^®^	Anacor Pharmaceuticals	PDE 4	Atopic dermatitis
Difamilast	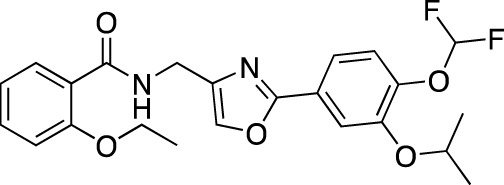	Moizerto^®^	Otsuka Pharmaceutical	PDE 4B	Atopic dermatitis
Drotaverine	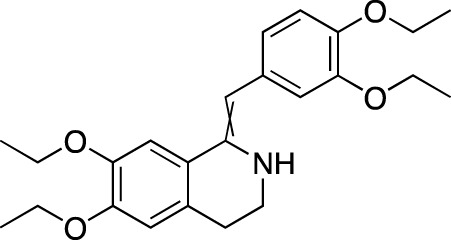	No-Spa^®^	Chinoin/Sanofi	PDE 4A	Spasm-induced functional bowel disorders
Ibudilast	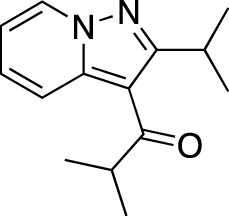	Ketas^®^, Eyevinal^®^	Kyorin Pharmaceutical	PDE 3A; PDE 4	Allergic conjunctivitis
Tofisopam	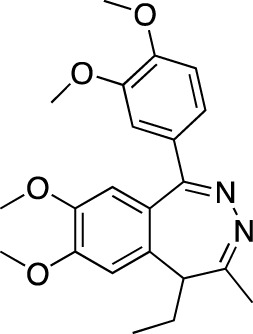	Emandaxin^®^, Grandaxin^®^	Egis Pharmaceuticals	PDE 2; PDE 3; PDE 4A; PDE 10	Anxiety

Recurrent aphthous ulcerations is a common chronic disease in the oral mucosa, characterized by solitary or multiple, recurrent, small ulcers with erythematous halos and yellow or gray pseudomembranes (Lau and Smith, 2022). Psoriasis is an immune-mediated genetic skin disease that can also manifest in joints; chronic plague psoriasis represents the majority of cases (Boehncke and Schön, 2015). Atopic dermatitis is a common inflammatory skin disorder of multifactorial etiology characterized by recurring and intensely pruritic lesions (Tollefson and Bruckner, 2014). In the skin, the PDE 4 enzymes have been shown to be primarily expressed in keratinocytes, neutrophils, Langerhans cells, and T-cells (Li et al., 2018). Additionally, peripheral blood mononuclear cells demonstrate higher PDE 4B and PDE 4D mRNA levels in psoriatic patients (Schafer et al., 2016). Earlier studies have also noted potential disturbances in adenylyl cyclase signalling, associated with psoriasis (Wright et al., 1973). In atopic dermatitis, overexpression and overactivity of PDE 4 enzymes leads to the production of inflammatory cytokines and imbalance of T-cell activity and polarization, causing skin inflammation and disease exacerbation (Li et al., 2018).

Clinically, amlexanox, apremilast, crisaborole, and difumilast have all showed positive safety and efficacy. Amlexanox, originally formulated as a 5% topical oral paste, was shown to accelerate complete ulcer healing and the time to resolution of pain, and reduce the ulcer size (Bell, 2005). Treatment with apremilast, according to the most recent Phase 3 trial in patients with recurrent psoriatic arthritis (*n* = 505 patients) (Edwards et al., 2016), resulted in 40% achieving 20% functional improvement (as compared to 18% taking placebo) and 41% achieving 50% reduction in skin involvement (vs. 24% taking placebo). As an orally active drug, its use can be associated with systemic ADRs, such as diarrhea, nausea, headache, upper respiratory tract infection, vomiting, nasopharyngitis, and abdominal pain (Cada et al., 2014). Additionally, its use is associated with a risk of depression and weight loss. Crisaborole, based on the results of two Phase 3 trials in atopic dermatitis (n_1_ = 759 patients, n_2_ = 763 patients) (Paller et al., 2016), has showed an improvement in skin involvement (3 2-grade improvement; 32.8% and 31.4% vs. 25.4% and 18.0% taking the vehicle-control), as well as a pruritus relieve. The only ADR associated with the treatment was application site pain (Paller et al., 2016). Difamilast, based on the results of a Phase 3 long-term study in the Japanese adult and pediatric patients (*n*_adult_ = 166 patients, *n*_pediatric_ = 200 patients) (Saeki et al., 2022), has shown a cumulative success EASI-75 (Eczema Area and Severity index) rates of 55.4% and 73.5%. The most common ADRs included dermatitis, acne and pigmentation disorder.

Here, PDE inhibitors are considered interesting additions to the established treatment strategies. Amlexanox is a first-line treatment option in apthous stomatitis (Lau and Smith, 2022). Apremilast is active in both psoriatic arthritis and cutaneous manifestations of the disease; however, its overall efficacy is low compared to other available options (Boehncke and Schön, 2015). Crisaborole is an option to treat mild-to-moderate disease, characterized by low systemic toxicity; it is reported, nevertheless, that it is not yet clear if it is characterized by lower toxicity than the other available topical therapeutic options (Li et al., 2018). Difamilast has showed attractive results in the Japanese population, more evidence is needed on patients of other ethnicities to discuss its wider use.

### Phosphodiesterase inhibitors in nervous system disorders

Three PDE inhibitors have been marketed in central and peripheral nervous system disorders (Table 4): drotaverine (No-Spa^®^), tofisopam (Grandaxin^®^) and caffeine (Cafcit^®^). Drotaverine is marketed in functional bowel disorders due to the smooth muscle spasm, while tofisopam is marketed in anxiety. Additionally, caffeine is approved by the FDA in 1999 for use in the apnea of prematurity.

Drotaverine, a structural analogue of papaverine, acts as a direct smooth muscle relaxant through PDE inhibition and Ca^2+^ channel blocking (Patai et al., 2016). Caffeine is a centrally acting methylxanthine with a complex mechanism of action; however, it is believed that the principal mechanism involves adenosine receptors antagonism, due to PDE inhibition requiring very high concentrations (Atik et al., 2017). Tofisopam is described as an atypical benzodiazepine that doesn’t interact with γ-aminobutyric acid receptors, but instead acts as a selective PDE inhibitor with the highest affinities to PDE 4A1 (0.42 μM) and PDE 10A1 (0.92 μM) (Rundfeldt et al., 2010).

The published clinical evidence on these drugs is generally scarce. Drotaverine had been studied as a spasmolytic in irritable bowel syndrome (IBS) and labor augmentation. In IBS, drotaverine was shown to improve abdominal symptoms, including pain frequency and severity, and stool frequency, based on the results of a double-blind placebo-controlled study (Rai et al., 2014). In labor augmentation, it reduced the duration of first and second stages of labor, but with a limited impact on pain (Singh et al., 2004). In apnea of prematurity, caffeine reduces the incidence of bronchopulmonary dysplasia and improves the rate of survival without neurodevelopmental disability in long-term use, in addition to reducing the frequency of apnea of prematurity and the need for mechanical ventilation during the first 7 days of therapy (Schmidt et al., 2006; Schmidt et al., 2007). On tofisopam, literature sources state that it is potent in alleviating vegetative symptoms accompanying anxiety disorders as an anxiolytic without sedative-hypnotic, muscle relaxant and anticonvulsive properties (Szegó et al., 1993).

To summarize, the nervous system disorders currently represent a niche use for PDE inhibitors. Drotaverine and tofisopam lack clinical evidence to discuss its wider applicability. Caffeine is being considered as the mainstay of apnea of prematurity pharmacotherapy; however, it is not considered to act primarily as a PDE inhibitor.

## Phosphodiesterase inhibitors in clinical research

In our dataset, we identified 52 PDE inhibitors which are either under ongoing investigation in clinical or pre-clinical studies, or which have been previously investigated but are either discontinued or have an unknown development status as of 2022. Among the agents in development (Tables 5, 7), indications are diverse, notably including CNS disorders, solid malignancies, metabolic, as well as inflammatory or immune-mediated disorders. Among the failed agents and/or agents in an unknown status (Table 6), the majority of indications are inflammatory disorders, CNS disorders, hematological and solid malignancies, and vascular disorders.

**TABLE 5:** Investigational PDE inhibitors in an ongoing clinical development. This table provides a summary on the novel PDE inhibitors that are currently investigated in clinical trials. The aggregated data on indications and clinical trials is listed as per studies identified on ClinicalTrials.gov, the EU Clinical Trials register and/or the developers’ websites. Chemical structures are derived from the PubChem database (Kim et al., 2021). Developer names are listed according to the relevant studies. Development status is listed according to the AdisInsight database., 2022 records^11^.

Drug name	Chemical structure	Developer	PDE selectivity	Indication(s)	Highest CT phase
MK-8189	Structure undisclosed	Merck Sharp & Dohme	PDE 10	Alzheimer’s disease (NCT05227118), schizophrenia (NCT05406440; NCT04624243)	Phase 2
BPN14770	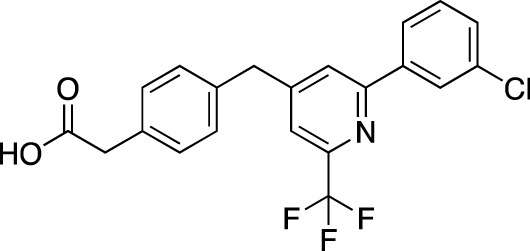	Tetra Therapeutics	PDE 4D	Alzheimer’s disease (NCT03817684), fragile X syndrome (NCT05163808; NCT05358886; NCT05367960)	Phase 2
CC-11050	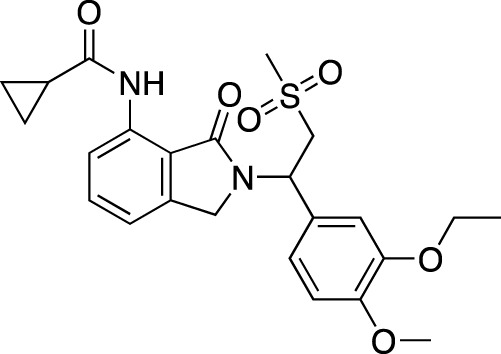	Celgene	PDE 4	Tuberculosis (NCT02968927), leprosy (NCT03807362), cutaneous lupus erythematosus (NCT01300208)	Phase 2
CTP-499	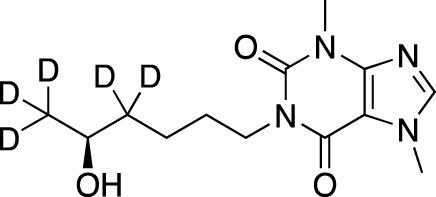	Concert Pharmaceuticals	PDE 4A, 4B	Diabetic nephropathy (NCT01487109), necrobiosis lipoidica	Phase 2
Ensifentrine	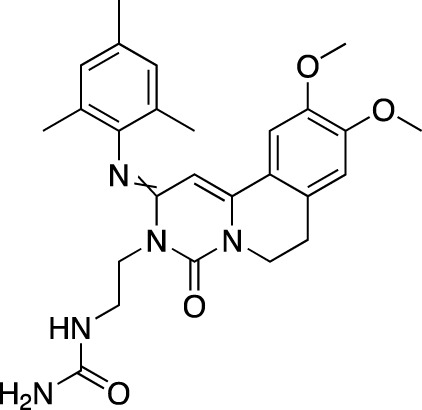	Vernalis/Verona Pharma	PDE 3A, 3B; PDE 4A, 4B	Chronic obstructive pulmonary disease, asthma, cystic fibrosis (NCT02919995), COVID-19 (NCT04527471)	Phase 3
HORA-PDE6B	Not applicable; a gene therapy product	Horama/Coave Therapeutics	PDE 6B	Retinitis pigmentosa (NCT03328130)	Phase 1/2
HT-0712	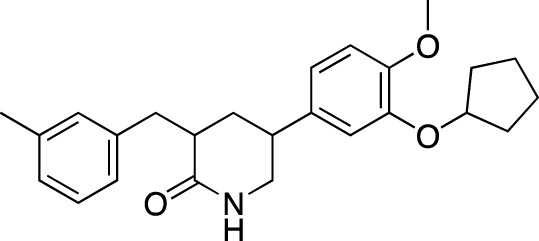	Inflazyme Pharmaceuticals/Dart Neurosciences	PDE 4B	Memory disorders (NCT02013310)	Phase 2
OMS527	Structure undisclosed	Asubio Pharma/Omeros	PDE 7	Addictions and compulsive disorders; movement disorders	Phase 1
PBF-999	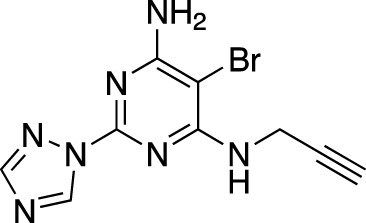	Palobiofarma	PDE 10	Advanced solid tumors (NCT03786484), Huntington’s disease (NCT02208934)	Phase 1
Tanimilast	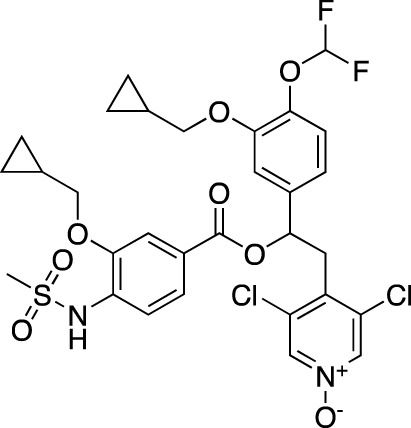	Chiesi Farmaceutici	PDE 4A, 4B	Allergic asthma, chronic obstructive pulmonary disease (NCT04636801; NCT04636814)	Phase 3
Tovinontrine	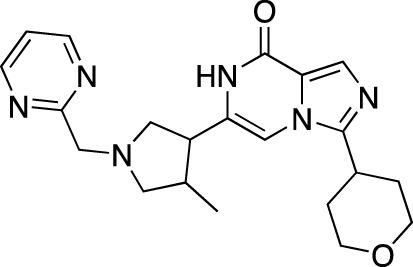	Lundbeck A/S/Imara	PDE 9	β-thalassemia, sickle cell anemia	Phase 2
Tipelukast	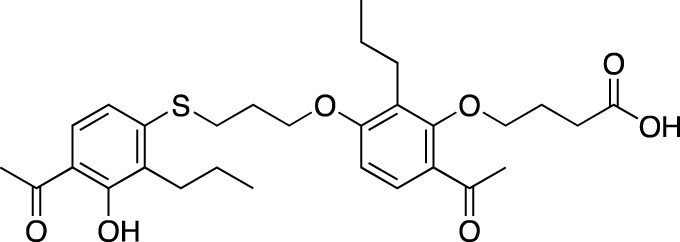	Kyorin Pharmaceutical/MediciNova	PDE 3A	Idiopathic pulmonary fibrosis (NCT02503657), non-alcoholic liver disease, hypertriglyceridemia, type 2 diabetes mellitus (NCT05464784)	Phase 2
ITI-214	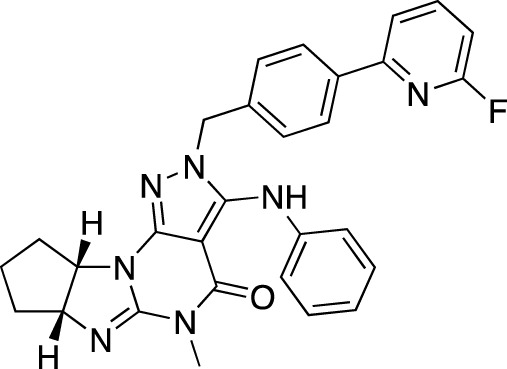	Intra-Cellular Therapies	PDE1	Heart failure (NCT03387215), Parkinson’s disease (NCT03257046)	Phase 1/2
BI-409306	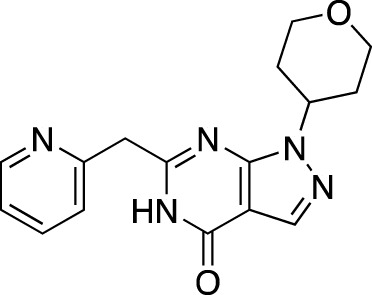	Boehringer Ingelheim	PDE 9	Alzheimer’s disease, schizophrenia (NCT03230097; NCT03351244)	Phase 2

**TABLE 6:** Investigational PDE inhibitors in discontinued or unknown clinical development. This table provides a summary on the investigational PDE inhibitors that have been evaluated clinically, but whose development is either discontinued as of 2022 or is otherwise unknown. The aggregated data on clinical trials is listed as per the ClinicalTrials.gov, the EU Clinical Trials register and PubMed databases and/or the developers’ websites. Chemical structures are derived from the PubChem database (Kim et al., 2021). Developer names are listed according to the relevant studies. Development status is listed according to the AdisInsight database records.

Drug name	Chemical structure	Developer	PDE selectivity	Indication(s)	Highest CT phase
Balipodect	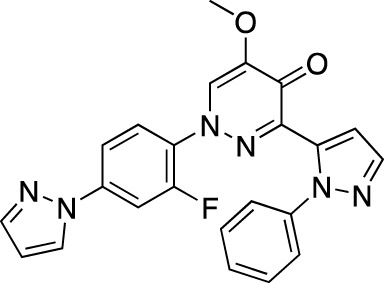	Takeda	PDE 10A	Schizophrenia	Phase 2
BLX-028914	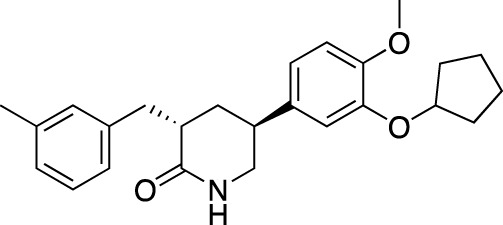	Dart NeuroSciences/Orexo	PDE4	Allergic rhinitis (NCT00758446)	Phase 2
CC 1088	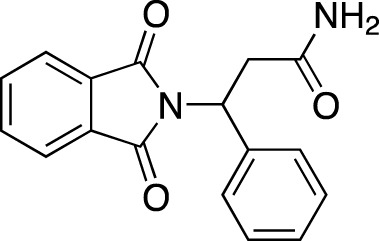	Celgene	PDE4	Myelodysplastic syndrome (NCT00045786), chronic lymphocytic leukemia (NCT00006097)	Phase 2
CI 1044	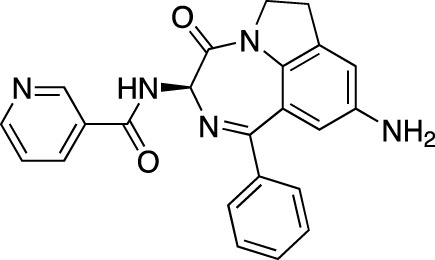	Pfizer	PDE4	Asthma, chronic obstructive pulmonary disease	Phase 1
Etazolate	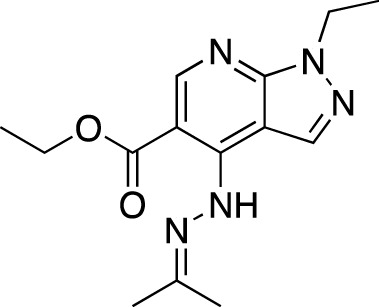	ExonHit Therapeutics	PDE4	Alzheimer’s disease; antipsychotic agent (NCT00880412)	Phase 2
EVP-6308	Structure undisclosed	EnVivo Pharmaceuticals	PDE 10	Schizophrenia (NCT02037074)	Phase 1
Exisulind	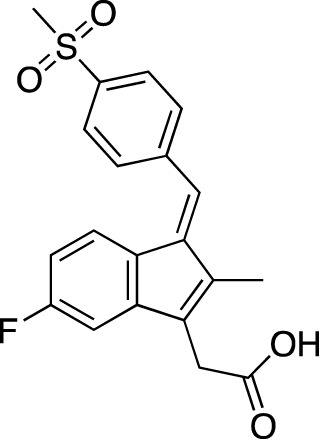	Osi Pharmaceuticals	PDE 2A; PDE 4D, 4C; PDE 5	Lung cancer (NCT00072618), prostate cancer (NCT00078910), gastrointestinal tumors (NCT00026468), breast cancer (NCT00037609; NCT00039520)	Phase 3
GSK256066	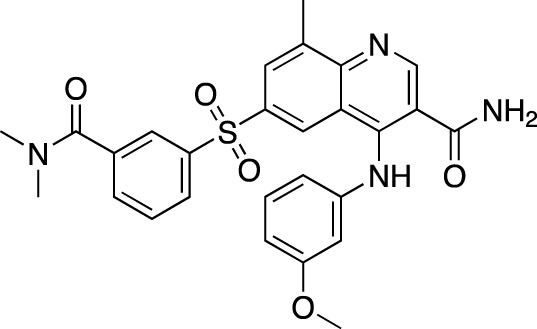	GlaxoSmithKline	PDE4	Allergic rhinitis (NCT00464568; NCT00612118; NCT00612820), asthma (NCT00380354), chronic obstructive pulmonary disease (NCT00549679)	Phase 2
IPL512,602	Structure undisclosed	Inflazyme Pharmaceuticals	PDE4	Asthma (NCT00330070)	Phase 2
IPL576,092	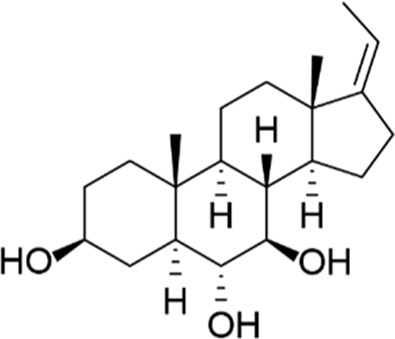	Inflazyme Pharmaceuticals	PDE4	Asthma	Phase 2
Mardepodect	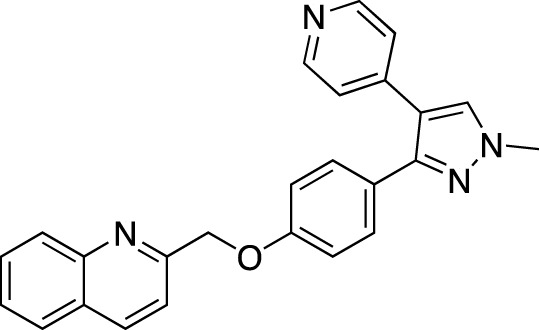	Pfizer	PDE 10	Schizophrenia, Huntington’s disease	Phase 2
MEM1414	Structure undisclosed	Memory Pharmaceuticals	PDE 4A, 4B	Asthma, Alzheimer’s disease	Phase 2
Oglemilast	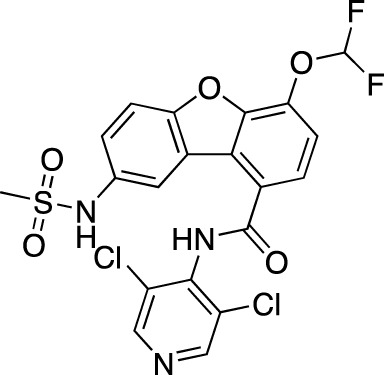	Glenmark Pharmaceuticals	PDE4	Asthma (NCT00322283; NCT00322686), chronic obstructive pulmonary disease (NCT00671073)	Phase 2
CEL-031	Structure undisclosed	OSI Pharmaceuticals/Celek Pharmaceuticals	PDE2, PDE5	Cancer, Crohn’s disease	Phase 2
OX914	Structure undisclosed	Orexo	PDE4A, 4B	Allergic rhinitis (NCT00758446), asthma	Phase 2
Parogrelil	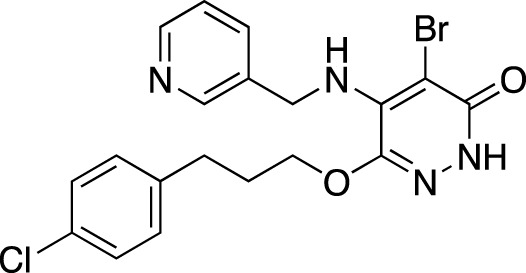	Nissan Chemical Industries	PDE3, PDE5	Asthma, intermittent claudication	Phase 2
Revamilast	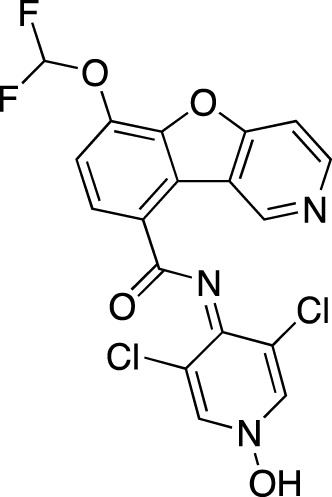	Glenmark Pharmaceuticals	PDE4	Asthma (NCT01436890), rheumatoid arthritis (NCT01430507)	Phase 2
Rolipram	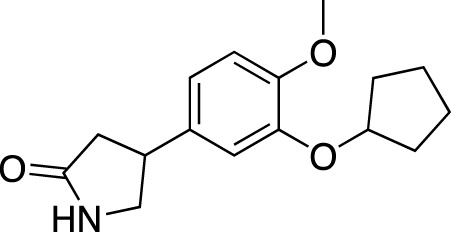	Bayer Schering Pharma	PDE 4B, 4D	Major depressive disorder (NCT00369798), Huntington’s disease (NCT01602900), multiple sclerosis (NCT00011375)	Phase 2
RO 20–1724	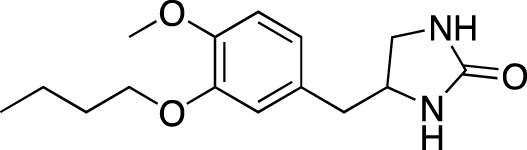	Roche	PDE4	Psoriasis	N/A
Ronomilast	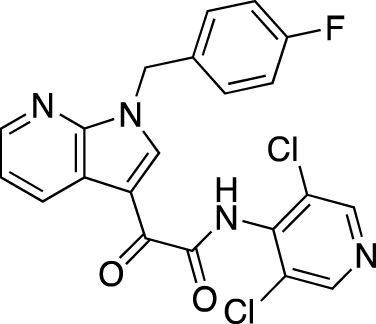	ASTA Medica	PDE4A, 4B	Chronic obstructive pulmonary disease	Phase 2
Tolafentrine	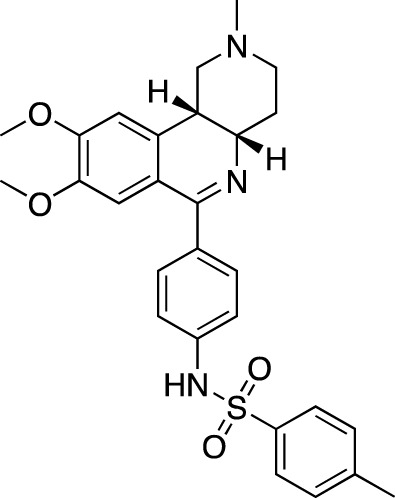	ALTANA Pharma	PDE3, PDE4	Asthma	N/A
Tetomilast	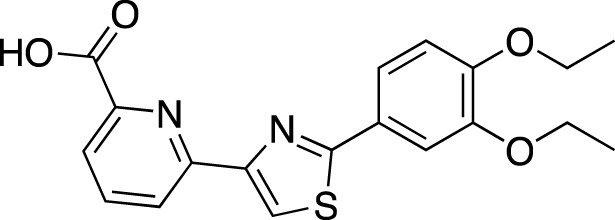	Otsuka Pharmaceutical	PDE 4	Crohn’s disease (NCT00317369; NCT00989573), ulcerative colitis (NCT00064454; NCT00092508); heart failure, reperfusion injury; chronic obstructive pulmonary disease (NCT00917150)	Phase 2
SLx-2101	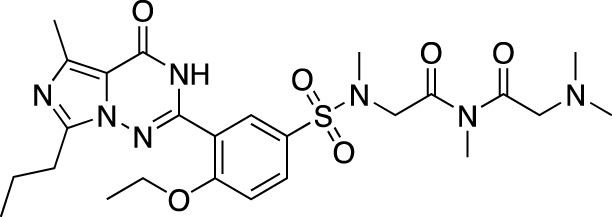	Surface Logix/Kadmon Pharmaceuticals	PDE5	Erectile dysfunction, Raynaud’s disease (NCT00528242), hypertension (NCT00562614; NCT00562549)	Phase 2
Zaprinast	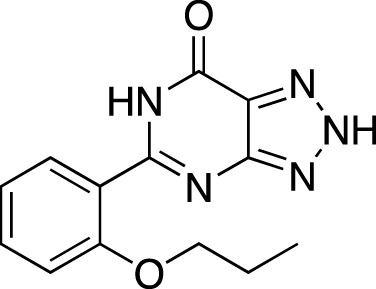	Aventis	PDE5, PDE6	Exercise-induced asthma; ischemic heart disease	N/A
OMS824	Structure undisclosed	Nura/Omeros	PDE 10	Huntington’s disease (NCT02074410), schizophrenia (NCT01952132)	Phase 2
DG071	Structure undisclosed	deCODE genetics	PDE4	Alzheimer’s disease	N/A

### Investigational phosphodiesterase inhibitors

Among the investigational CNS disorders, neurodegenerative and psychiatric conditions, such as Alzheimer’s disease (AD) and schizophrenia, are particularly prevalent. In schizophrenia, PDE 10A inhibition is reported as a plausible approach due to the expression pattern of PDE 10A in striatal medium spiny neurons and functional similarity of its inhibition to the dopamine D_2_ receptor antagonism (Snyder and Vanover, 2017). These agents include MK-8189, TAK-063 (also known as balipodect), FRM 6308 (EVP-6308), MP-10 (mardepodect) and OMS 824. Out of all these agents, published evidence on clinical studies exists for MK-8189 and TAK-063; both agents have showed acceptable tolerability, but the efficacy was reported to be low (Krogmann et al., 2019). As of 2022, only MK-8189 appears to be in the ongoing development, with a Phase 1 multiple ascending dose study of safety, tolerability, pharmacokinetics and the effect on QTc currently in the Recruiting stage (NCT05406440). In addition to PDE 10A, at least one PDE 9A inhibitor is undergoing development in psychiatric and neurodegenerative disorders, BI-409306 (osoresnontrine). The drug is currently in an Ongoing Phase 1 trial of the effect on ketamine-induced cognitive deficit in healthy male subjects (NCT04602221). It was previously investigated in cognitive impairment due to schizophrenia (Brown et al., 2019) and prodromal-to-mild AD (Frölich et al., 2019), but failed to demonstrate efficacy.

In AD, PDE4 inhibitors represent the bulk of investigational approaches (Klyucherev et al., 2022). The rationale here stems from the effect of cAMP on memory formation and cognition; an intracellular increase in cerebral cAMP activates protein kinase A associated with cAMP response element binding protein which is vital for synaptic plasticity (Tibbo et al., 2019). The drugs include BPN14770 (putative generic name zatolmilast), HT-0712, EHT 202 (etazolate), MEM1414, and DG071. BPN14770 has so far been investigated in three Phase 1 trials (NCT02648672, NCT02840279, and NCT03030105) in AD and was found to be associated with an improvement in working memory in healthy elderly subjects at low-to-mid doses, as reported in the developer’s press release (Tetra Discovery, 2016)^8^. Its ongoing development, however, goes predominantly in the direction of fragile X syndrome, a genetic neurodevelopmental disorder, characterized by intellectual disability, hyperactivity, sensory hypersensitivity, autistic-like behavior and susceptibility to seizures (Gurney et al., 2019). According to the results of a placebo-controlled Phase 2 study, BPN14770 resulted in a significant cognitive improvement related to language and improvements in caregiver scales rating language and daily functioning (Berry-Kravis et al., 2021). Additionally, there is one Recruiting Phase 2/3 study in male adolescents (NCT05163808), a Phase 3 Not yet recruiting study in male adults (NCT05358886) and an open-label extension Phase 3 Not yet recruiting study to assess the long-term safety and tolerability in subjects of the former studies. HT-0712 was reported to improve long-term memory in mice (Bourtchouladze et al., 2003; Peters et al., 2014) and facilitate behavioral recovery and cortical reorganization after an ischemic insult (MacDonald et al., 2007); however, no results from a clinical study could be identified. EHT 202 was investigated in a Phase 2 study as an adjunct to an acetylcholinesterase inhibitor in patients with mild-to-moderate AD (NCT00880412); no results had been published. Another CNS indication under ongoing investigation is Parkinson’s disease (PD); a previously mentioned PDE 1 inhibitor ITI-214 has shown reduction in neuroinflammation and enhancement of memory performance and cognition in preclinical models (Snyder et al., 2016; Pekcec et al., 2018; O’Brien et al., 2020). Clinically, it was reported to improve the motor functions, according to a Phase 1/2 trial in patients with mild to moderate PD (Davis et al., 2019).

Another notable investigational indication are neoplasms which is developing rapidly across numerous targets (reviewed in Attwood et al., 2021; Sokolov et al., 2021). In cancer, the PDE overexpression is observed in numerous types of cancer and its inhibition has shown positive effects on preclinical models (Peng et al., 2018). Antineoplastic PDE inhibitors in clinical development include PBF-999, exisulind, CEL-031 and CC-1088. PBF-999, a dual adenosine A2a receptor antagonist/PDE 10 inhibitor, is investigated in a Phase 1 Recruiting trial (NCT03786484) in patients with immunotherapy-naïve and pretreated advanced solid tumors. According to the developer’s website (PaloBioPharma Pipeline, 2022)^9^, colon cancer appears to be the primary indication. Exisulind is the sulfone derivative of sulindac, a selective apoptotic antineoplastic drug (Exisulind: Aptosyn, 2004). It had been evaluated in a wide range of tumors, the most recent being prostate (Weight et al., 2012), lung (Govindan et al., 2009), and melanoma (Curiel-Lewandrowski et al., 2012); however, no further development could be identified, possibly due to insufficient efficacy. CEL-031 is another apoptotic antineoplastic drug, developed for bladder cancer^10^; lack in its recent development could be due to poor oral bioavailability. CC-1088, a thalidomide analogue, was originally developed for myelodysplastic syndromes (Dredge, 2005). According to an *in vitro* study on myeloma cells (Molostvov et al., 2004), CC- 1088 acts by inhibiting production of vascular endothelial growth factor and interleukin (IL)- 6, and by inducing apoptosis^11^.

Among the other investigational indications of note are blood disorders, *retinitis pigmentosa*, *necrobiosis lipoidica*, autoimmune diseases, mycobacterial diseases, the novel coronavirus disease (COVID-19) and metabolic diseases. In blood disorders, tovinontrine is evaluated for sickle cell anemia in a Phase 2a Ongoing study to evaluate the long-term safety, tolerability and pharmacodynamics (NCT04053803). A gene therapy using an adeno-associated virus vector AAV2/5-hPDE6 is in a Phase 1/2 Ongoing trial (NCT03328130) in patients with retinitis pigmentosa. CC-11050 is currently evaluated in a Phase 2 Recruiting study in patients with leprosy and *erythema nodosum leprosum* (NCT03807362). The drug has also been studied in cutaneous *lupus erythematosus*, tuberculosis (TB) and HIV infection; published evidence in TB suggests that PDE inhibition can enhance the lung function recovery (Subbian et al., 2016; Wallis et al., 2021) and improve responsiveness to the antibacterial therapy (Subbian et al., 2016). In HIV, CC-11050 was shown to have no impact on CD4 counts or plasma viremia, but led to a decrease in natural killer cells and plasma IL-8 level, as well as to an increase in IL-6 (Boulougura et al., 2019). In COVID-19, ensifentrine had been investigated in a Phase 2 study to evaluate the efficacy and safety in the recovery of patients hospitalized with the disease (NCT04527471); the study is in an Unknown status since 2021, however. Nevertheless, tanimilast, another PDE 4 inhibitor, was found to exert a modulatory effect on the SARS-CoV-2 genomic ssRNA-induced pro-inflammatory and Th1- polarising potential of dendritic cells (Nguyen et al., 2022). MN-001 (tipelukast) was previously investigated in idiopathic pulmonary fibrosis (Phase 2, no results published) and currently is in a Not yet recruiting Phase 2 study in patients with non-alcoholic fatty liver disease, type 2 diabetes mellitus (T2DM), and hypertriglyceridemia (NCT05464784).

### Experimental phosphodiesterase inhibitors

In addition to the PDE inhibitors in clinical development, mentioned above, we have identified several experimental drugs. Among the novel indications here is Duchenne muscular dystrophy, potentially manageable using a PDE 4 and PDE 5 inhibitor combination therapy. According to an *in vivo* study of a novel PDE 4 RP 73401 (piclamilast) in the mdx mice, it reduces the mRNA level of profibrotic genes in the gastrocnemius and diaphragm of the animal, and significantly reduced the percentage affected by fibrosis (Nio et al., 2017).

Many of experimental agents are PDE 10 inhibitors. JNJ-42314415, MR 1916, SEP-39, and ASP9436 are novel PDE 10 inhibitors with an antipsychotic activity; characterization of JNJ- 42314415, in particular, have showed that the effects of PDE 10A inhibition against dopaminergic stimulants and on catalepsy were potentiated by a D_1_ antagonist, providing a potential strategy to improve safety profile of PDE 10A inhibitors through reducing dopamine D_2_ and concomitantly potentiating dopamine D_1_ receptor-mediated neurotransmission (Megens et al., 2014). In addition, MR-1916, when combined with risperidone, resulted in a significant enhancement of the conditioned avoidance response in rats without affecting extrapyramidal side effects (Arakawa and Maehara, 2020).

Several agents target novel PDE targets. BRL-50481, a PDE 7 inhibitor, have showed neuroprotector (Chen et al., 2020) and anti-inflammatory properties (Kim et al., 2022) on animal models. Lu AF33241, a dual PDE 2A/PDE 10A inhibitor, was found to attenuate sub-chronic phencyclidine-induced deficits in novel object recognition and displayed antipsychotic-like activity (Redrobe et al., 2015). EHNA, a PDE 2 inhibitor, was recently shown to inhibit proliferation and stimulate migration of human osteosarcoma cells (Murata et al., 2019) and to inhibit growth and invasion of human malignant melanoma cells (Hiramoto et al., 2014). Interestingly, a PDE 3/4 inhibitor zardaverine was recently shown to act as a potent cytotoxic agent in embryonal rhabdomyosarcoma and cervical carcinoma cell lines (Cartledge et al., 2017), as well as in hepatocellular carcinoma (Sun et al., 2014); the mechanism, however, is independent of PDE inhibition.

One more agent to discuss is resveratrol, a naturally occurring polyphenol marketed as a dietary supplement due to its antioxidant properties (Salehi et al., 2018). Several preclinical studies have showed that some of its biological properties are mediated *via* PDE inhibition. Resveratrol was found to impart neuroprotection in brain ischemia by inhibiting PDEs and regulating the cAMP/AMPK/SIRT1 pathway, which reduces ATP energy consumption (Wan et al., 2016). Additionally, it was found to enhance glucose-stimulated insulin secretion by pancreatic β-cells (Rouse et al., 2014) and ameliorate aging-associated metabolic disorders by multiple mechanisms, including an increase in mitochondrial biogenesis and protection against diet-induced obesity and glucose intolerance (Park et al., 2012).

Several studies involving resveratrol were done on human subjects as well, such as AD, Gulf War syndrome, T2DM, postmenopausal osteoporosis and COVID-19. In AD, it was found to alter the Aβ40 levels and the brain volume loss increase (Turner et al., 2015), decrease MMP9 levels, modulate neuro-inflammation and induce adaptive immunity (Moussa et al., 2017); the mechanism involved may not be related to the PDE inhibition, however. In Gulf War syndrome, a chronic multi-symptom disorder of an unknown etiology that affects veterans of the Gulf War, it reduced the symptom severity (Hodgin et al., 2021). In T2DM, a systematic review of three trials has reported a neutral impact on glycosylated hemoglobin A1c (mean difference 0.1% vs. placebo), fasting blood glucose levels (2 mg/dl difference vs. placebo), and insulin resistance (−0.35 difference vs. placebo) (Jeyaraman et al., 2020). In postmenopausal osteoporosis, resveratrol has showed the potential to slow bone loss in the lumbar spine and femoral neck (Wong et al., 2020). In COVID-19, resveratrol was shown to reduce angiotensin-converting enzyme 2 expression in the adipose tissue, a potential SARS-CoV-2 reservoir contributing to massive viral spread in COVID-19 patients with obesity (de Ligt et al., 2021). The direction and status of overall clinical development for this compound is uncertain, however; among the major barriers reported for its development is poor bioavailability and solubility (Sun et al., 2014).

## Discussion

Here, we provide a comprehensive study of the clinical trials and FDA approved drugs in relationship to their therapeutic targets. As of 2022, the majority of commercially available PDE inhibitors are marketed in cardiovascular disorders and inflammatory diseases, offering valuable therapeutic options. Particularly, PDE 5 inhibitor are the first-line option in ED, while PDE 4 inhibitors are actively explored in inflammatory conditions of the skin and COPD, showing variable, but promising results. The ongoing clinical research moves towards diversification with CNS disorders being particularly prevalent, however, specifically schizophrenia and AD. Other interesting directions are mycobacterial infectious diseases, such as tuberculosis and leprosy, and potential applicability in COVID-19. The clinical evidence on efficacy is currently limited and often conflicting, however. Many of the investigational PDE inhibitors, particularly drugs investigated in asthma or COPD as an indication are either discontinued, suspended or are in an otherwise unknown status. A common reason seems to be lack of efficacy and/or unsatisfactory ADR profile; however, there are gaps in published evidence on most agents, making it difficult to assess the issue.

Among the marketed agents, current issues include compliance-limiting ADRs and often insufficient clinical efficacy. A potential solution to the lack of efficacy could be directing drug development efforts to evaluate combinatory regimens, as suggested for PDE 5 inhibitors in ED; combining PDE 5 inhibitors with other therapeutic approaches, such as antioxidants, showed improved outcomes without increasing ADRs (Mykoniatis et al., 2021). A similar strategy is suggested for PDE 4 inhibitors in inflammatory diseases (Li et al., 2018). The issue of compliance-limiting ADRs could be amended by adjusting prescribing regimens and/or *via* rational drug design based on evidence of the effect of structural features on safety. Additionally, increasing the isoform specificity and/or designing allosteric modulators is mentioned in the literature as a plausible strategy to improve the ADR profile and efficacy (Li et al., 2018; Chen and Yan, 2021).

PDE enzymes are a long-established pharmacological target and the extensive research involving these proteins contributes to a continuous interest towards further expansion of the PDE inhibitors’ clinical use. The future development continues to diversify, particularly thanks to the experimental agents and/or agents in the preclinical development (Table 7). We expect this trend to continue in future. Among the particularly interesting and promising novel indications is tuberculosis, given how challenging the treatment can be and the prevalence of the disease. CNS disorders, such as schizophrenia and AD, are another interesting direction; however, there are recognized gaps in understanding the molecular mechanisms of psychiatric (Menniti et al., 2021) and neurodegenerative disorders (Tibbo et al., 2019) that need to be filled in order to maximize the CNS drug development efforts. Neoplasms are another important direction; several PDE inhibitors have shown antitumor activity, but there is a continuous need for better understanding of PDE molecular biology (Peng et al., 2018) for this direction to gain traction.

**TABLE 7:** Selected experimental PDE inhibitors. This table provides a summary on some of the experimental PDE inhibitors in pre-clinical studies. The aggregated data on studies is listed as per the PubMed database. Chemical structures are derived from the PubChem database (Kim et al., 2021). Developer names are listed according to the relevant studies.

Drug name	Chemical structure	Developer	PDE selectivity	Indication(s)
ASP9436	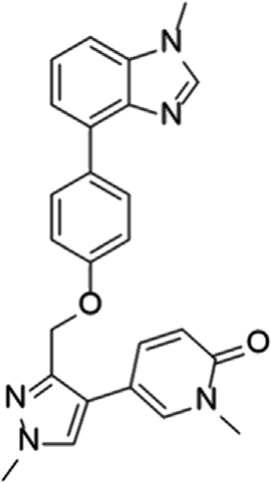	Astellas Pharma	PDE 10	Schizophrenia (Hamaguchi et al., 2015)
BAY-73–6691	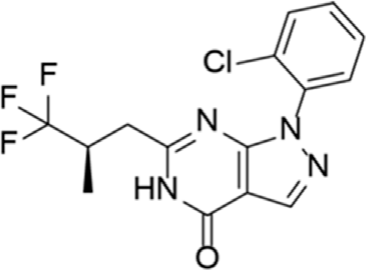	Bayer	PDE 9	Alzheimer’s disease (Li et al., 2018)
BRL-50481	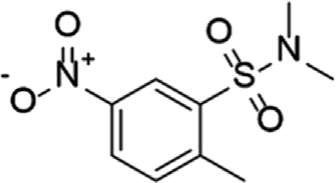	GlaxoSmithKline	PDE 7	CNS disorders (Chen et al., 2020), inflammation (Kim et al., 2022)
CC-3052	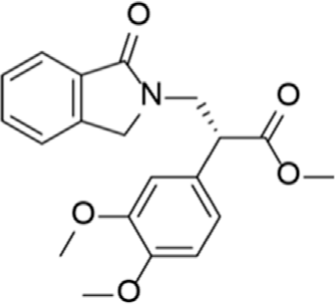	Celgene	PDE 4	Tuberculosis (Subbian et al., 2016), HIV (Guckian et al., 2000; La Maestra et al., 2000)
EHNA	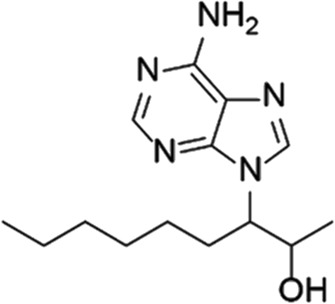	N/A	PDE 2	Cancer (Bernard et al., 2014; Hiramoto et al., 2014; Murata et al., 2019), HIV (Sei et al., 1989)
JNJ-42314415	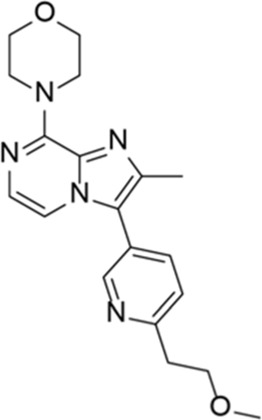	Janssen Pharmaceutica	PDE 10	Schizophrenia (Megens et al., 2014)
Lu AF33241	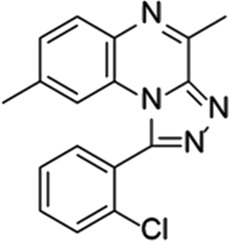	Lundbeck A/S	PDE 2/10	CNS disorders (Redrobe et al., 2015)
MR1916	Structure undisclosed	Mochida Pharmaceutical	PDE 10	Schizophrenia, Parkinson disease (Arakawa & Maehara, 2020; Arakawa and Maehara., 2020)
RP 73401	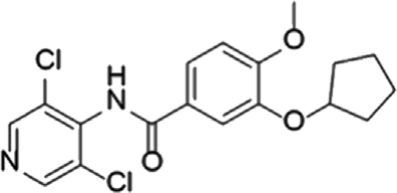	Aventis	PDE 4	Inflammation, Duchenne muscular dystrophy (Nio et al., 2017)
SEP39	Structure undisclosed	Sunovion Pharmaceuticals	PDE 10	CNS disorders (Jones et al., 2015)
Zardaverine	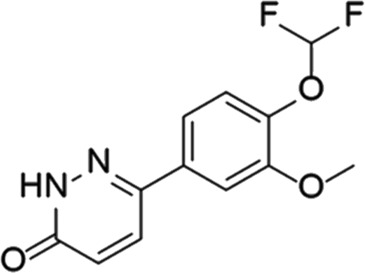	ALTANA Pharma	PDE 3, PDE 4	Asthma (Kips et al., 1993), cancer (Sun et al., 2014; Cartledge et al., 2017)
Resveratrol	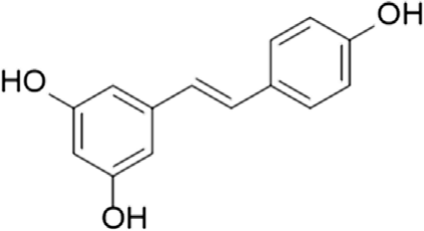	N/A	PDE 4B, 4D	Alzheimer’s disease, Friedreich’s ataxia, metabolic disorders, type 2 diabetes mellitus, depression, Gulf War syndrome, polycystic ovarian syndrome, chronic obstructive pulmonary disease

The global market of PDE inhibitors is currently driven predominantly by the PDE 5 inhibitors and the unmet clinical needs and the increase in ED prevalence is also considered to contribute (Transparency Market Research, 2021). It is expected to grow by USD 2.86 billion throughout 2022–2026 period (Technavio, 2022), with the compound annual growth rate (CAGR) of 5.81%. During the 2022–2027 forecast period the market CAGR is expected to be 6.2% (Market Data Forecast, 2022). The ongoing COVID-19 pandemic has impacted the market, at least partially due to the clinical uses of PDE inhibitors in diseases that exacerbate the novel SARS-CoV-2 infection, such as CVDs and obstructive pulmonary diseases (Market Data Forecast, 2022). One of the largest challenges to the market’s growth can be considered the negative ADR profile that contributes to the lack of FDA and EMA approvals (Market Data Forecast, 2022).

## Conclusion

Here, we provide a comprehensive overview of PDE inhibitors with focus on novel agents and indications. The current clinical uses of PDE inhibitors include COPD, vascular and cardiovascular disorders and skin inflammatory disorders. There is a trend for diversification of clinical uses for PDE inhibitors with numerous novel agents being in clinical and preclinical development. While a significant portion of drugs under development have not been successful, the scientific community recognizes some of the present gaps in knowledge that had been slowing the development. The continuous efforts to develop and evaluate both the approved and novel agents will undoubtedly expand our understanding of the PDE pharmacology and will likely contribute to the further growth.

## Footnotes

1
https://www.centerwatch.com

2
https://www.accessdata.fda.gov/scripts/cder/daf/index.cfm

3
https://www.ema.europa.eu/en/medicines

4
https://www.nice.org.uk/guidance/published?ngt=Technology%20appraisal%20guidance&ndt=Guidance

5
https://clinicaltrials.gov/ct2/home

6
https://www.clinicaltrialsregister.eu/ctr-search/search

7
https://www.who.int/news-room/fact-sheets/detail/cardiovascular-diseases-(cvds)

8
https://tetratherapeutics.com/wp-content/uploads/2016/11/FINAL-Tetra-Phase-1-121616-FINAL.pdf

9
https://www.palobiofarma.com/pipeline-2/

10
https://www.businesswire.com/news/home/20111222005044/en/Celek-Pharmaceuticals-Receives-National-Cancer-Institute-Award-to-Develop-CEL-031-for-the-Treatment-of-Bladder-Cancer

11
https://adisinsight.springer.com
